# Author Correction: Octadecanoids as emerging lipid mediators in cnidarian-dinoflagellate symbiosis

**DOI:** 10.1038/s42003-026-09970-8

**Published:** 2026-04-06

**Authors:** Marina T. Botana, Robert E. Lewis, Alessandro Quaranta, Olivier Salamin, Johanna Revol-Cavalier, Clint A. Oakley, Ivo Feussner, Mats Hamberg, Arthur R. Grossman, David J. Suggett, Virginia M. Weis, Craig E. Wheelock, Simon K. Davy

**Affiliations:** 1 School of Biological Sciences, Victoria University of Wellington, Wellington, New Zealand; 2 Unit of Integrative Metabolomics, Institute of Environmental Medicine, Karolinska Institutet, Stockholm, Sweden; 3 Larodan Research Laboratory, Karolinska Institutet, Stockholm, Sweden; 4 Department of Plant Biochemistry, Albrecht-von-Haller-Institute for Plant Sciences and Goettingen Center for Molecular Biosciences (GZMB), University of Goettingen, Goettingen, Germany; 5 Carnegie Institution for Science, Department of Plant Biology, Stanford, CA USA; 6 KAUST Coral Restoration Initiative (KCRI) and Division of Biological and Environmental Science and Engineering (BESE), King Abdullah University of Science and Technology, Thuwal, Saudi Arabia; 7 Department of Integrative Biology, Oregon State University, Corvallis, OR USA; 8 Department of Respiratory Medicine and Allergy, Karolinska University Hospital, Stockholm, Sweden

**Keywords:** Chemical ecology, Fatty acids, Fatty acids

Correction to: *Communications Biology* 10.1038/s42003-025-09104-6, published online 04 November 2025

In the original version of the article, the bottom left panel of Fig. 5b (13-HOTE) was accidentally a duplicated version of the bottom left panel of Fig. 5a. This has been corrected in the HTML and PDF versions of the article.

