# Supplementary material for: Octadecanoids as emerging lipid mediators in cnidarian-dinoflagellate symbiosis
Source: Commun Biol. 2025 Nov 4;8:1519. doi: 10.1038/s42003-025-09104-6 (PMC12586643; doi:10.1038/s42003-025-09104-6)
Supplement: Supplementary file 2 — Supplementary Information [file 42003_2025_9104_MOESM2_ESM.pdf]

Supplementary Data

Octadecanoids as emerging lipid mediators in the cnidarian-dinoflagellate symbiosis

Marina T. Botana[1,2], Robert E. Lewis[1], Alessandro Quaranta[2], Olivier Salamin[2], Johanna Revol-Cavalier[2,3], Clint A. Oakley[1], Ivo Feussner[4], Mats Hamberg[2,3], Arthur R. Grossman[5], David J. Suggett[6], Virginia M. Weis[7], Craig E. Wheelock[2,8,*], Simon K. Davy[1,*]

[1]School of Biological Sciences, Victoria University of Wellington, Wellington 6012, New Zealand

[2]Unit of Integrative Metabolomics, Institute of Environmental Medicine, Karolinska Institutet, 171 77 Stockholm, Sweden.

[3]Larodan Research Laboratory, Karolinska Institutet, 171 65 Stockholm, Sweden.

[4]Department of Plant Biochemistry, Albrecht-von-Haller-Institute for Plant Sciences and Goettingen Center for Molecular Biosciences (GZMB), University of Goettingen, 37077 Goettingen, Germany

[5]Carnegie Institution for Science, Department of Plant Biology, Stanford, CA 94 305, USA

[6]KAUST Coral Restoration Initiative (KCRI) and Division of Biological and Environmental Science and Engineering (BESE), King Abdullah University of Science and Technology, Thuwal 23 955, Saudi Arabia

[7]Department of Integrative Biology, Oregon State University, Corvallis, OR 97331, USA

[8]Department of Respiratory Medicine and Allergy, Karolinska University Hospital, 171 76 Stockholm, Sweden.

*co-corresponding authors

Craig Wheelock
Unit of Integrative Metabolomics
Institute of Environmental Medicine
Karolinska Institutet
171 77 Stockholm, Sweden
craig.wheelock@ki.se

Simon Davy
School of Biological Sciences
Victoria University of Wellington
Wellington 6012, New Zealand
simon.davy@vuw.ac.nz

Table of contents

Figures

Supplementary Figure 1. Cell density curves of *Breviolum minutum* and *Durusdinium trenchii* monocultures…………………………………………………………………………....4

Supplementary Figure 2: Sequence alignment of new *Breviolum minutum* (Bm) putative LOX with other 13(*S*) (At LOX3) from *Arabidopsis thaliana*. ……………………………..…...5

Supplementary Figure 3: Supplementary Figure 3: Sequence alignment of new *Durisdinium trenchii* (Dt) putative LOXes with other 13(*S*) (At LOX3) from *Arabidopsis thaliana*. …………...7

Supplementary Figure 4: Active expression representation of all novel putative LOX candidates in Symbiodiniaceae……………………...…………….………………………………12

Tables

Supplementary Data 1: Quantification of octadecanoids presented as ng/g dry biomass

Supplementary Data 2: Relative amounts of *R* and *S* enantiomers and calculated **ee** values for octadecanoids in average sample groups

Supplementary Data 3: One-way ANOVA with pairwise Tukey *post hoc* test results

Supplementary Data 4: Octadecanoids that differed in the host with a fold-change >2 and p < 0.05

Supplementary Data 5: Octadecanoids that differed in the symbiont with a fold-change >2 and p < 0.05

Supplementary Data 6: Species confirmation of symbionts from cultures and symbiotic anemones

Supplementary Data 7: Description and analytical information about internal standards and new octadecanoids that were added to the original platform

Supplementary Data 8: Genomic stramenopile and alveolate protein references used for Blastp ORF homology

Supplementary Data 9: GenBank access numbers for phylogeny tree and protein sequency homology

Supplementary Data 10: NCBI access numbers for RNA-seq libraries used for novel LOX candidate gene expression counts

Supplementary Data 11: GenBank access numbers for phylogeny tree and protein sequency homology

NOTE: The data for Supplementary Data 1-11 is provided in a separate Excel file

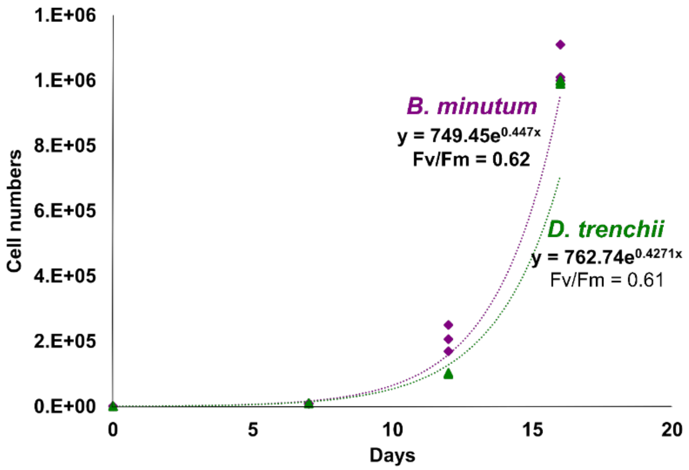

Supplementary Figure 1: Cell density curves of *Breviolum minutum* and *Durusdinium trenchii* monocultures (n=3). Biomass was sampled after 16 days when both species were in exponential growth phase; photosynthetic efficiency (F_v/F_m) was also measured at this point. Replicate values were fitted to an exponential curve for the estimation of growth rates.

Alignment of novel LOX sequence for *Breviolum minutum* with *A. thaliana* LOXes

```
AtLOX3            MALAKELMGYPLITERSSLVSSASHFKKRTQSTQFSINPFDRRPRKTKSGVVAAISEDLV   60

AtLOX1            ------------------------------------------------------MF----   2

AtLOX5            -----------------------------------------------------MIHTDIA   7

Bm_Mf_1.05b.01    ------------------------------------------------------------   0


AtLOX3            KTLRFSTTTGDR--KSEEEEKAAVKFKVRAVVTVRNKNKEDLKETLVKHLDAFADKIGRN   118

AtLOX1            ---------GELRDLLTGGGNETTTKKVKGTVVLMKKNVLDFNDFNASFLDRLHEFLGNK   53

AtLOX5            EILCVKPKTTKKTKTMEEDVKKTTTMKIEGEVVVMKKNLLDFKDVMASLLDRVNELLGRR   67

Bm_Mf_1.05b.01    ------------------------------------------------------------   0


AtLOX3            IVLELISTQLDPKT--KLPKKSNAAVLKDWSKKSKT--KAERVHYTAEFTVDAAFGSPGA   174

AtLOX1            ITLRLVSSDVTDSENGSKGKLGKAAHLEDWITTIT-SLTAGESAFKVTFDYETDFGYPGA   112

AtLOX5            VSLHLISSHQPDPANEKRGRLGKAAHLEKWVTKIKTSVTAEETAFGVTFDWDESMGPPAA   127

Bm_Mf_1.05b.01    ------------------------------------------------------------   0


AtLOX3            ITVMNKHQKEFFLESITIEGFA-----LGPVHFPCNSWVQSQKDHPDKRIFFTNQPYLPN   229

AtLOX1            FLIRNSHFSEFLLKSLTLEDVP----GHGRVHYICNSWIYPAKHYTTDRVFFSNKTYLPH   168

AtLOX5            FVIKNHHHSQFYLKSLTLRGFPDGEGGATAIHFICNSWIYPNHRYRSDRVFFSNKAYLPS   187

Bm_Mf_1.05b.01    ------------------------------------------------------------   0


AtLOX3            ETPSGLRVLREKELKNLRGDG-SGVRKLSDRIYDFDVYNDLGNPDKSSELSRPKLGGK-E   287

AtLOX1            ETPATLLKYREEELVSLRGTG-EGELKEWDRVYDYAYYNDLGVPPKNP---RPVLGGTQE   224

AtLOX5            ETPELIKELREEELKNLRGNEKGGEFKEWDRVYDYAYYNDLGAPDKGPDSVRPVLGGSPE   247

Bm_Mf_1.05b.01    ------------------------------------------------------------   0


AtLOX3            VPYPRRCRTGRQSTVSDKDAESRVE--KPLPMYVPRDEQFEESKQDTFAAGRLKAVLHHL   345

AtLOX1            YPYPRRGRTGRKPTKEDPQTESRLPITSSLDIYVPRDERFGHLKMSDFLAYALKAIAQFI   284

AtLOX5            LPYPRRGKTGRKSTKSDPKSESRLALL-NLNIYVPRDERFSHVKFSDFLAYALKSVTQVL   306

Bm_Mf_1.05b.01    ------------------------------------------------------------   0


AtLOX3            IPSLKASI--VAEDFADFGEIDRLYKEGLLLKLGFQDDIFKKFPLPKVVVDTLQESTKGL   403

AtLOX1            QPALEAVFDDTPKEFDSFEDVLKIYEEGIDLPNQALIDSIVKNIPLEMLKEIFRTDGQKF   344

AtLOX5            VPEIASVCDKTINEFDSFEDVFHLYDGSIKLANGHTISKLRDVIPWEMFRELVRNDGERF   366

Bm_Mf_1.05b.01    ------------------------------------------------------------   0
```

```
AtLOX3          LKYDTPKILSKDKNAWLRDDEFARQAIAGINPVNIERVKTFPPVSNLDPKIYGPQHSALT  463
AtLOX1          LKFPVPQVIKEDKTAWRTDEEFAREMLAGLNPVVIQLLKEFPPKSKLDSESYGNQNSTIT  404
AtLOX5          LKYPLPDILKESRSAWRTDEEFAREMLAGLNPVVISRLQEFPPKSCLDSAKYGNQHSSIR  426
Bm_Mf_1.05b.01  ------------------------------------------------------------  0

AtLOX3          DDHIIGHLDGFSVQQALEENRLYMLDYHDIFLPFLDRINALDGRKAYATRTIFFLTRLGT  523
AtLOX1          KSHIEHNLDGLTVEEALEKERLFILDHHDTLMPYLGRVNTTT-TKTYASRTLLFLKDDGT  463
AtLOX5          TEHIESNMNGLNVQEALEQNKLYILDHHDALMPYLTRINSTN-TKTYATRTLLLLQADGT  485
Bm_Mf_1.05b.01  ------------------------------------------------------------  0

AtLOX3          LKPVAIELSLPPHGPKH--R---SKRVLTPPVDATSNWMWQLAKAHVSSNDAGVHQLVNH  578
AtLOX1          LKPLVIELSLPHPNGDKFGA---VSEVYTPGE-GVYDSLWQLAKAFVGVNDSGNHQLISH  519
AtLOX5          LKPLAIELSLPHAQGESYGS---VSKVFTPAEKGVEGSVWQLAKAYAAVNDSGYHQLISH  542
Bm_Mf_1.05b.01  -M------IIDQGRGPLVEDFEQKSLVVKPQ-----DDDWERSKFRFRSSLFSLVTLVDH  48

AtLOX3          WLRTHACLEPF-ILAAHRQLSAMHPIFKLLDPHMRYTLEINALARQSLISADGVIEGGFT  637
AtLOX1          WMQTHASIEPF-VIATNRQLSVLHPVFKLLEPHFRDTMNINALARQILINGGGIFEITVF  578
AtLOX5          WLQTHAVIEPF-IIASNRQLSVVHPIHKLLHPHFRDTMNINALARHVLINSDGVLERTVF  601
Bm_Mf_1.05b.01  LYFLHLQLANFFVTSLREQMAEYHPIRRFLTPFTYYTISVNDNAAKNLVAQRSMGPRCFA  108

AtLOX3          AGAYGMEMSAAAYKSSWRFDMEGLPADLI------------RRGMAIPDATQPHGLKLLI  685
AtLOX1          PSKYAMEMSSFIYKNHWTFPDQALPAELK------------KRGMAVEDPEAPHGLRLRI  626
AtLOX5          PSRYAMEMSSSIYK-NWVFTEQALPKDLL------------KRGVAVEDPNSDNGVKLLI  648
Bm_Mf_1.05b.01  LTDKGFNL-------AFAAAPHLQVWGYEVPAEEGGPFLNLKNYFA------WKRAKG--  153

AtLOX3          EDYPYANDGLLLWSAIQTWVRTYVERYYPNPNL----IKTDSELQSWYSESIN------V  735
AtLOX1          KDYPYAVDGLEVWYAIESWVRDYIFLFYKIEED----IQTDTELQAWWKEVRE------E  676
AtLOX5          EDYPFAVDGLEIWSAIKTWVTEYCTFYYNNDKT----VQTDTEIQSWWTELRT------K  698
Bm_Mf_1.05b.01  --------VACFWLI---------------------------------------------  160

AtLOX3          GHADLRDADWWPELSTVDDLVSIL---TTLIWLASAQHAALNFGQYPYGGYVPNRPPLMR  792
AtLOX1          GHGDKKSEPWWPKMQTREELVESC---TIIIWVASALHAAVNFGQYPVAGYLPNRPTISR  733
AtLOX5          GHGDKRHESWWPSMQTRDDLIETC---TIIIWIASALHAAVNFGQYPYAGFLPNRPTVSR  755
Bm_Mf_1.05b.01  ------------------------------------------------------------  160
```

```
AtLOX3          RLIPDESDPEYASFISHPEKYYFSSMPSLAQTSKFMAVVDTLSTHSPDEEYIGERQQPSI   852
AtLOX1          QYMPKENTPEFEELEKNPDKVFLKTITAQLQTLLGISLIEILSTHSSDEVYLGQRDSK-E   792
AtLOX5          RFMPEPGTDEYAELEEDADVAFLKTITPQLQTLLGISIIEILSMHSTDEIYLGQRDSP-N   814
Bm_Mf_1.05b.01  ------------------------------------------------------------   160

AtLOX3          WTG---------DAEIVEAFYGFAAEIGRIEKEIEKRNADPDRRNRCGAGVLPYELLVPS   903
AtLOX1          WAA---------EKEALEAFEKFGEKVKEIEKNIDERNDDETLKNRTGLVKMPYTLLFPS   843
AtLOX5          WTA---------DDEPLEAFKRFGKELELIENNIIRRNNDKRFKNRTGPVNIPYTLLYPN   865
Bm_Mf_1.05b.01  ------------------------------------------------------------   160

AtLOX3          -----SEPGVTCRGVPNSVSI   919
AtLOX1          -----SEGGVTGRGIPNSVSI   859
AtLOX5          TTDYTREGGITGKGIPNSVSI   886
Bm_Mf_1.05b.01  ---------------------   160
```

Supplementary Figure 2: Sequence alignment of new *Breviolum minutum* (Bm) putative LOX with other 13(*S*) (At LOX3) from *Arabidopsis thaliana*. According to the alignment, the sequence is likely a 13*S*-LOX, defined by the Hornung (yellow/green) and Coffa (blue) sites (Wasternack & Feussner, 2018). The sequence is partial, and it was not found in any publicly available dataset. *See Excel file in electronic version.*

Alignment of novel LOX sequences for *Durusdinium trenchii* and their complete sequences from GenBank with *A. thaliana* LOXes

```
Dt_CCMP2556_1   ------------------------------------------------------------   0
Dt_CCMP2556_2   ------------------------------------------------------------   0
CAK8999115.1    ------------------------------------------------------------   0
CAK9070246.1    ------------------------------------------------------------   0
AtLOX3          MALAKELMGYPLITERSSLVSSASHFKKRTQSTQFSINPFDRRPRKTKSGVVAAISEDLV   60
AtLOX1          ------------------------------------------------------MF----   2
AtLOX5          -----------------------------------------------------MIHTDIA   7

Dt_CCMP2556_1   ------------------------------------------------------------   0
Dt_CCMP2556_2   ------------------------------------------------------------   0
CAK8999115.1    ------------------------------------------------------------   0
```

```
CAK9070246.1        ------------------------------------------------------------   0
AtLOX3              KTLRFSTTTGDR--KSEEEEKAAVKFKVRAVVTVRNKNKEDLKETLVKHLDAFADKIGRN   118
AtLOX1              ---------GELRDLLTGGGNETTTKKVKGTVVLMKKNVLDFNDFNASFLDRLHEFLGNK   53
AtLOX5              EILCVKPKTTKKTKTMEEDVKKTTTMKIEGEVVVMKKNLLDFKDVMASLLDRVNELLGRR   67

Dt_CCMP2556_1       ------------------------------------------------------------   0
Dt_CCMP2556_2       ------------------------------------------------------------   0
CAK8999115.1        ------------------------------------------------------------   0
CAK9070246.1        ------------------------------------------------------------   0
AtLOX3              IVLELISTQLDPKT--KLPKKSNAAVLKDWSKKSKT--KAERVHYTAEFTVDAAFGSPGA   174
AtLOX1              ITLRLVSSDVTDSENGSKGKLGKAAHLEDWITTIT-SLTAGESAFKVTFDYETDFGYPGA   112
AtLOX5              VSLHLISSHQPDPANEKRGRLGKAAHLEKWVTKIKTSVTAEETAFGVTFDWDESMGPPAA   127

Dt_CCMP2556_1       ------------------------------------------------------------   0
Dt_CCMP2556_2       ------------------------------------------------------------   0
CAK8999115.1        ------------------------------------------------------------   0
CAK9070246.1        ------------------------------------------------------------   0
AtLOX3              ITVMNKHQKEFFLESITIEGFA-----LGPVHFPCNSWVQSQKDHPDKRIFFTNQPYLPN   229
AtLOX1              FLIRNSHFSEFLLKSLTLEDVP----GHGRVHYICNSWIYPAKHYTTDRVFFSNKTYLPH   168
AtLOX5              FVIKNHHHSQFYLKSLTLRGFPDGEGGATAIHFICNSWIYPNHRYRSDRVFFSNKAYLPS   187

Dt_CCMP2556_1       ------------------------------------------------------------   0
Dt_CCMP2556_2       ------------------------------------------------------------   0
CAK8999115.1        ------------------------------------------------MEQSPLIGESLY   12
CAK9070246.1        ------------------------------------------------MEQSPLIGESLY   12
AtLOX3              ETPSGLRVLREKELKNLRGDG-SGVRKLSDRIYDFDVYNDLGNPDKSSELSRPKLGGK-E   287
AtLOX1              ETPATLLKYREEELVSLRGTG-EGELKEWDRVYDYAYYNDLGVPPKNP---RPVLGGTQE   224
AtLOX5              ETPELIKELREEELKNLRGNEKGGEFKEWDRVYDYAYYNDLGAPDKGPDSVRPVLGGSPE   247

Dt_CCMP2556_1       ------------------------------------------------------------   0
Dt_CCMP2556_2       ------------------------------------------------------------   0
CAK8999115.1        A--------------------------PSWPVVQPPEPSFYSLGCCQTCLRIPAAFGCGG   46
CAK9070246.1        A--------------------------PSWPVVQPPEPSFYSLGCCQTCLRIPAAFGCGG   46
AtLOX3              VPYPRRCRTGRQSTVSDKDAESRVE--KPLPMYVPRDEQFEESKQD--------------   331
AtLOX1              YPYPRRGRTGRKPTKEDPQTESRLPITSSLDIYVPRDERFGHLKMS--------------   270
```

```
AtLOX5          LPYPRRGKTGRKSTKSDPKSESRLALL-NLNIYVPRDERFSHVKFS--------------   292

Dt_CCMP2556_1   ------------------------------------------------------------   0
Dt_CCMP2556_2   ------------------------------------------------------------   0
CAK8999115.1    CRICCYSILAVL-------------------LFALLLGFYPIVVALVLVLLLLLGLPISY   87
CAK9070246.1    CRICCYSILAVL-------------------LFALLLGFYPIVVALVLVLLLLLGLPISY   87
AtLOX3          -TFAAGRLKAVLHHLIPSLKASI--VAEDFADFGEIDRLYKEG------LLLKLGFQD--   380
AtLOX1          -DFLAYALKAIAQFIQPALEAVFDDTPKEFDSFEDVLKIYEEG------IDLPNQALI--   321
AtLOX5          -DFLAYALKSVTQVLVPEIASVCDKTINEFDSFEDVFHLYDGS------IKLANGHTI--   343

Dt_CCMP2556_1   ------------------------------------------------------------   0
Dt_CCMP2556_2   ------------------------------------------------------------   0
CAK8999115.1    AMIYLLFLNPPPELAWKIAKLTQLLRLAFYQFPKPA-------EWRVDQLAPKGPLETGL   140
CAK9070246.1    AMIYLLFLNPPPELAWKIAKLTQLLRLAFYQFPKPA-------EWRVDQLAPKGPLETGL   140
AtLOX3          ---DIFKKFPLPKVVVDT---LQESTKGLLKYDTPKILSKDKNAWLRDDEFARQAI-AGI   433
AtLOX1          ---DSIVKNIPLEMLKEI---FRTDGQKFLKFPVPQVIKEDKTAWRTDEEFAREML-AGL   374
AtLOX5          ---SKLRDVIPWEMFREL---VRNDGERFLKYPLPDILKESRSAWRTDEEFAREML-AGL   396

Dt_CCMP2556_1   ------------------------------------------------------------   0
Dt_CCMP2556_2   ------------------------------------------------------------   0
CAK8999115.1    LQYAIAIRVPFLQQDELYAGGLFERFLRSSPSTEFVTSSVFST----LP----QMELKDM   192
CAK9070246.1    SQYAIAIRVPFLQQDELYAGGLFERFLRSSPSTEFVTSSVFST----LP----QMELKDM   192
AtLOX3          NPVNIERV-------------------KTFPPVSNLDPKIYGPQHSALTDDHIIGHLDGF   474
AtLOX1          NPVVIQLL-------------------KEFPPKSKLDSESYGNQNSTITKSHIEHNLDGL   415
AtLOX5          NPVVISRL-------------------QEFPPKSCLDSAKYGNQHSSIRTEHIESNMNGL   437

Dt_CCMP2556_1   ------------------------------------------------------------   0
Dt_CCMP2556_2   ------------------------------------------------------------   0
CAK8999115.1    SLFKEGENPVEYVMGVVQDIYPRINQEWTDKTSDRALTHLCLHGLGAHRLERADSTHPGC   252
CAK9070246.1    SLFKEGENPVEYVMGVVQDIYPRINQEWTDKTSDRALTHLCLHGLGAHRLERADSTHPGC   252
AtLOX3          SVQQALEENRLYMLD--------------------------YHDIFLPFLDRINALDGRK   508
AtLOX1          TVEEALEKERLFILD--------------------------HHDTLMPYLGRVNTTT-TK   448
AtLOX5          NVQEALEQNKLYILD--------------------------HHDALMPYLTRINSTN-TK   470

Dt_CCMP2556_1   -------------MPRSPISQGNDVYFDKDFRVVKIVR------FENGEVWED-QKITTF   40
```

```
Dt_CCMP2556_2     -------------MPRSPISQGNDAYFDKDFRVVKIVR------FENGEVWED-QKITTF   40
CAK8999115.1      SYVVRTNQLSTLPVREGYETYGNDVYFDKDFRVVKIVR------FENGEVWED-QKITTF   305
CAK9070246.1      SYVVRTNQLSTLPVREGYETYGNDAYFDKDFRVVKIVR------FENGEVWED-QKITTF   305
AtLOX3            AYATRTI------------------FFLTRLGTLKPVAIELSLPPHGPKH--RSKRVLTP   548
AtLOX1            TYASRTL------------------LFLKDDGTLKPLVIELSLPHPNGDKFGAVSEVYTP   490
AtLOX5            TYATRTL------------------LLLQADGTLKPLAIELSLPHAQGESYGSVSKVFTP   512

Dt_CCMP2556_1     LPDGS--RDWEYAKFCFRCSLFTLVTLVDHLYGTHLQLANVGVQAMREQLSVDHPVRRFL   98
Dt_CCMP2556_2     LPDGS--RDWEYAKFCFRCSLFTLVTLVDHLYGTHLQLANVGVQAMREQLSVDHPVRRFL   98
CAK8999115.1      LPDGS--RDWEYAKFCFRCSLFTLVTLVDHLYGTHLQLANVGVQAMREQLSVDHPVRRFL   363
CAK9070246.1      LPDGS--RDWEYAKFCFRCSLFTLVTLVDHLYGTHLQLANVGVQAMREQLSVDHPVRRFL   363
AtLOX3            PVDATSNWMWQLAKAHVSSNDAGVHQLVNHWLRTHACLEPF-ILAAHRQLSAMHPIFKLL   607
AtLOX1            GE-GVYDSLWQLAKAFVGVNDSGNHQLISHWMQTHASIEPF-VIATNRQLSVLHPVFKLL   548
AtLOX5            AEKGVEGSVWQLAKAYAAVNDSGYHQLISHWLQTHAVIEPF-IIASNRQLSVVHPIHKLL   571

Dt_CCMP2556_1     VPFSYGSININDLARTTLVTRDSWLPRAVALDDEGLQ-------LAWASAFQILPPDYIT   151
Dt_CCMP2556_2     VPFSYGSININDLARTTLVTRDSWLPRAVALDDEGLQ-------LAWASAFQILPPDYIT   151
CAK8999115.1      VPFSYGSININDLARTTLVTRDSWLPRAVALDDEGLQ-------LAWASAFQILPPDYIT   416
CAK9070246.1      VPFSYGSININDLARTTLVTRDSWLPRAVALDDEGLQ-------LAWASAFQILPPDYIT   416
AtLOX3            DPHMRYTLEINALARQSLISADGVIEGGFTAGAYGMEMSAAAYKSSWRFDMEGLPADLIR   667
AtLOX1            EPHFRDTMNINALARQILINGGGIFEITVFPSKYAMEMSSFIYKNHWTFPDQALPAELKK   608
AtLOX5            HPHFRDTMNINALARHVLINSDGVLERTVFPSRYAMEMSSSIYK-NWVFTEQALPKDLLK   630

Dt_CCMP2556_1     NESDPIKMLESLFDREAQIEKKR-SEGL-----FTAYYKQALQYWKILHGFVSSYLDHYY   205
Dt_CCMP2556_2     NESDPIKMLESLFDREAQIEKKR-SEGL-----FTAYYKQALQYWKILHGFVSSYLDHYY   205
CAK8999115.1      NESDPIKMLESLFDREAQIEKKR-SEGL-----FTAYYKQALQYWKILHGFVSSYLDHYY   470
CAK9070246.1      NESDPIKMLESLFDREAQIEKKR-SEGL-----FTAYYKQALQYWKILHGFVSSYLDHYY   470
AtLOX3            RGM--------------AIPDATQPHGLKLLIEDYPYANDGLLLWSAIQTWVRTYVERYY   713
AtLOX1            RGM--------------AVEDPEAPHGLRLRIKDYPYAVDGLEVWYAIESWVRDYIFLFY   654
AtLOX5            RGV--------------AVEDPNSDNGVKLLIEDYPFAVDGLEIWSAIKTWVTEYCTFYY   676

Dt_CCMP2556_1     GTGAKGDLAMAADQELKLFILQAINLVQTLASPLTGHRVNELWPHLSDARKRRIMTNFIT   265
Dt_CCMP2556_2     GTGAKGDLAMAADQELKLFILQAINLVQTLASPLTGHRVNELWPHLSDARKRRIMTNFIT   265
CAK8999115.1      GTGAKGDLAMAADQELKLFILQAINLVQTLASPLTGHRVNELWPHLSDARKRRIMTNFIT   530
CAK9070246.1      GTGAKGDLAMAADQELKLFILQAINLVQTLASPLTGHRVNELWPHLSDARKRRIMTNFIT   530
```

AtLOX3	PNPNL----IKTDSELQSWYSESIN------VGHADLRDADWWPELSTVDDLVSIL---T	760
AtLOX1	KIEED----IQTDTELQAWWKEVRE------EGHGDKKSEPWWPKMQTREELVESC---T	701
AtLOX5	NNDKT----VQTDTEIQSWWTELRT------KGHGDKRHESWWPSMQTRDDLIETC---T	723
Dt_CCMP2556_1	RFCELSTAGHEQVGDVQ----AY--------------AQDPSFCSFSWPKSLRREGALVA	307
Dt_CCMP2556_2	RFCELSTAGHEQVGDVQ----AY--------------AQDPSFCSFSWPKSLRREGALVA	307
CAK8999115.1	RFCELSTAGHEQVGDVQ----AY--------------AQDPSFCSFSWPKSLRREGALVA	572
CAK9070246.1	RFCELSTAGHEQVGDVQ----AY--------------AQDPSFCSFSWPKSLRREGALVA	572
AtLOX3	TLIWLASAQHAALNFGQYPYGGYVPNRPPLMRRLIPDESDPEYASFISHPEKYYFSSMPS	820
AtLOX1	IIIWVASALHAAVNFGQYPVAGYLPNRPTISRQYMPKENTPEFEELEKNPDKVFLKTITA	761
AtLOX5	IIIWIASALHAAVNFGQYPYAGFLPNRPTVSRRFMPEPGTDEYAELEEDADVAFLKTITP	783
Dt_CCMP2556_1	PKEVGLGVALIMALT--STPMPRLLVREPTDDWSHLFPGASEADRSKLNSIFERFQSELQ	365
Dt_CCMP2556_2	PKEVGLGVALIMALT--STPMPRLLVREPTDDWSHLFPGASEADRSKLNSIFERFQSELQ	365
CAK8999115.1	PKEVGLGVALIMALT--STPMPRLLVREPTDDWSHLFPGASEADRSKLNSIFERFQSELQ	630
CAK9070246.1	PKEVGLGVALIMALT--STPMPRLLVREPTDDWSHLFPGASEADRSKLNSIFERFQSELQ	630
AtLOX3	LAQTSKFMAVVDTLSTHSPDEEYIGERQQPSIWTG---------DAEIVEAFYGFAAEIG	871
AtLOX1	QLQTLLGISLIEILSTHSSDEVYLGQRDSK-EWAA---------EKEALEAFEKFGEKVK	811
AtLOX5	QLQTLLGISIIEILSMHSTDEIYLGQRDSP-NWTA---------DDEPLEAFKRFGKELE	833
Dt_CCMP2556_1	TFSKEC--	371
Dt_CCMP2556_2	TFSKECD---	372
CAK8999115.1	TFSKECDDYNAEAKNRAFPNDFGLWVFN-----PK-------------YLETSVSI	668
CAK9070246.1	TFSKECDDYNAEAKNRAFPNDFGLWVFN-----PK-------------YLETSVSI	668
AtLOX3	RIEKEIEKRNADPDR---RNRCGAGVLPYELLVPS-----SEPGVTCRGVPNSVSI	919
AtLOX1	EIEKNIDERNDDETL---KNRTGLVKMPYTLLFPS-----SEGGVTGRGIPNSVSI	859
AtLOX5	LIENNIIRRNNDKRF---KNRTGPVNIPYTLLYPNTTDYTREGGITGKGIPNSVSI	886

Supplementary Figure 3: Sequence alignment of new *Durisdinium trenchii* (Dt) putative LOXes with other 13(*S*) (At LOX3) from *Arabidopsis thaliana*. According to the alignment, the sequences are most likely 9*S*-LOXes, as defined by the Hornung (yellow/green) and Coffa (blue) sites (Wasternack & Feussner, 2018). The complete sequences for *D. trenchii* were found in GenBank (CAK8999115.1 and CAK9070246.1) annotated as "unnamed proteins" and again aligned with the same *A. thaliana* LOXes. *See Excel file in electronic version.*

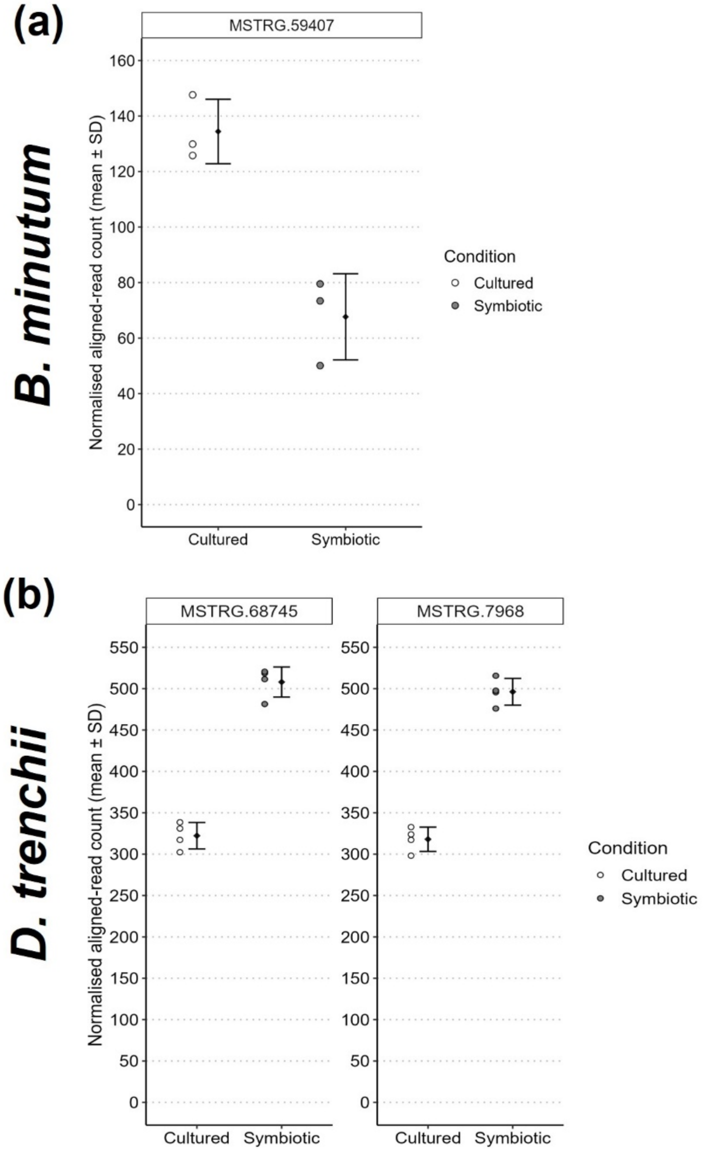

Supplementary Figure 4: Active expression representation of all novel putative LOX candidates in Symbiodiniaceae. Error bars represent standard deviation (SD). Different expression of genes between conditions were tested using DeSeq2 as explained in Methods with p-adjusted < 0.001. **(a)** For *B. minutum*, of the 38,797 expressed genes to pass the multi-sample filtering, MSTRG.59407 had a mean normalized expression across all samples (baseMean; n = 6) of 101.04 reads, ranking it 13,801st in the 64th percentile of expressed genes; **(b)** For *D. trenchii*, of the 20,717 expressed genes to pass the multi-sample filtering, MSTRG.7968 and MSTRG.68745 had mean normalized expression counts (baseMean, n = 8) of 407.14 and 415.14, respectively. MSTRG.7968 and MSTRG.68745 ranked 17,060th and 17,148th in expression within the 18th and 17th percentile of expressed genes, respectively.

Supplementary Data – see Excel File "Supplementary Data 2"

Supplementary Data 1: Quantification of octadecanoids presented as ng/g dry biomass. *See Excel file in electronic version.*

Supplementary Data 2: Relative amounts of *R* and *S* enantiomers and calculated **ee** values for octadecanoids in average sample groups. Values suggesting enzymatic biosynthesis are highlighted. *See Excel file in electronic version.*

Supplementary Data 3: One-way ANOVA with pairwise Tukey *post hoc* test results. Only compounds that were statistically significant ($p < 0.05$) with FDR are listed. *See Excel file in electronic version.*

Supplementary Data 4: Octadecanoids that differed in the host with a fold-change >2 and $p < 0.05$. Pairwise comparisons were between symbiotic hosts, colonized with *Breviolum minutum* (Host_B) and *Durusdinium trenchii* (Host_D), and *versus* the aposymbiotic host (Host_Apo). *See Excel file in electronic version.*

Supplementary Data 5: Octadecanoids that differed in the symbiont with a fold-change >2 and $p < 0.05$. Pairwise comparisons were between the symbionts *Breviolum minutum* (B_symbiotic) and *Durusdinium trenchii* (D_symbiotic) when in symbiosis, and when isolated in culture (B_cultured and D_cultured, respectively). *See Excel file in electronic version.*

Supplementary Data 6: Species confirmation of symbionts from cultures and symbiotic anemones. *See Excel file in electronic version*.

Supplementary Data 7: Internal standards and additional octadecanoids that were included in the current work that are not described in the original method of Quaranta *et al*. (2022). *See Excel file in electronic version.*

Supplementary Data 8: Concentration of internal standards mix spiked into samples. *See Excel file in electronic version.*

Supplementary Data 9: Genomic stramenopile and alveolate protein references used for Blastp ORF homology filtering of *Breviolum minutum* and *Durusdinium trenchii* transcriptomes. *See Excel file in electronic version.*

Supplementary Data 10: NCBI access numbers for RNA-seq libraries used for novel LOX candidate gene expression counts. *See Excel file in electronic version.*

Supplementary Data 11: GenBank access numbers for phylogeny tree and protein sequency homology. *See Excel file in electronic version.*
