# Supplementary material for: Octadecanoids as emerging lipid mediators in cnidarian-dinoflagellate symbiosis
Source: Commun Biol. 2025 Nov 4;8:1519. doi: 10.1038/s42003-025-09104-6 (PMC12586643; doi:10.1038/s42003-025-09104-6)
Supplement: Supplementary file 3 — Description of Additional Supplementary Materials [file 42003_2025_9104_MOESM3_ESM.pdf]

Description of Additional Supplementary Files

File name: Supplementary Data 1

Description: Quantification of octadecanoids presented as ng/g dry biomass

File name: Supplementary Data 2

Description: Relative amounts of R and S enantiomers and calculated ee values for octadecanoids in average sample groups. Values suggesting enzymatic biosynthesis are highlighted.

File name: Supplementary Data 3

Description: One-way ANOVA with pairwise Tukey post hoc test results. Only compounds that were statistically significant ($p < 0.05$) with FDR are listed.

File name: Supplementary Data 4

Description: Octadecanoids that differed in the host with a fold-change >2 and $p < 0.05$. Pairwise comparisons were between symbiotic hosts, colonized with Breviolum minutum (Host_B) and Durusdinium trenchii (Host_D), and versus the aposymbiotic host (Host_Apo).

File name: Supplementary Data 5

Description: Octadecanoids that differed in the symbiont with a fold-change >2 and $p < 0.05$. Pairwise comparisons were between the symbionts Breviolum minutum (B_symbiotic) and Durusdinium trenchii (D_symbiotic) when in symbiosis, and when isolated in culture (B_cultured and D_cultured, respectively).

File name: Supplementary Data 6

Description: Species confirmation of symbionts from cultures and symbiotic anemones.

File name: Supplementary Data 7

Description: Internal standards and additional octadecanoids that were included in the current work that are not described in the original method of Quaranta et al. (2022).

File name: Supplementary Data 8

Description: Concentration of internal standards mix spiked into samples.

File name: Supplementary Data 9

Description: Genomic stramenopile and alveolate protein references used for Blastp ORF homology filtering of Breviolum minutum and Durusdinium trenchii transcriptomes.

File name: Supplementary Data 10

Description: NCBI access numbers for RNA-seq libraries used for novel LOX candidate gene expression counts.

File name: Supplementary Data 11

Description: GenBank access numbers for phylogeny tree and protein sequency homology.
