# Supplementary material for: Octadecanoids as emerging lipid mediators in cnidarian-dinoflagellate symbiosis
Source: Commun Biol. 2025 Nov 4;8:1519. doi: 10.1038/s42003-025-09104-6 (PMC12586643; doi:10.1038/s42003-025-09104-6)
Supplement: Supplementary file 5 — Reporting Summary [file 42003_2025_9104_MOESM5_ESM.pdf]

nature portfolio

Corresponding author(s): COMMSBIO-24-8635

Last updated by author(s): Oct 19, 2025

Reporting Summary

Nature Portfolio wishes to improve the reproducibility of the work that we publish. This form provides structure for consistency and transparency in reporting. For further information on Nature Portfolio policies, see our Editorial Policies and the Editorial Policy Checklist.

Statistics

For all statistical analyses, confirm that the following items are present in the figure legend, table legend, main text, or Methods section.

n/a	Confirmed	
☐	☒	The exact sample size (*n*) for each experimental group/condition, given as a discrete number and unit of measurement
☐	☒	A statement on whether measurements were taken from distinct samples or whether the same sample was measured repeatedly
☐	☒	The statistical test(s) used AND whether they are one- or two-sided<br>*Only common tests should be described solely by name; describe more complex techniques in the Methods section.*
☐	☒	A description of all covariates tested
☐	☒	A description of any assumptions or corrections, such as tests of normality and adjustment for multiple comparisons
☐	☒	A full description of the statistical parameters including central tendency (e.g. means) or other basic estimates (e.g. regression coefficient) AND variation (e.g. standard deviation) or associated estimates of uncertainty (e.g. confidence intervals)
☐	☒	For null hypothesis testing, the test statistic (e.g. *F*, *t*, *r*) with confidence intervals, effect sizes, degrees of freedom and *P* value noted<br>*Give P values as exact values whenever suitable.*
☒	☐	For Bayesian analysis, information on the choice of priors and Markov chain Monte Carlo settings
☒	☐	For hierarchical and complex designs, identification of the appropriate level for tests and full reporting of outcomes
☒	☐	Estimates of effect sizes (e.g. Cohen's *d*, Pearson's *r*), indicating how they were calculated

Our web collection on statistics for biologists contains articles on many of the points above.

Software and code

Policy information about availability of computer code

Data collection	No software was used for data collection.
Data analysis	Fastp, RNA-Seq aligner Spliced Transcripts Alignment to a Reference (STAR version 2.7.10b), StringTie version 2.0.3, Transdecoder predict version 5.7, hmmscan v3.4, Rsubread v2.16.1, DeSeq2, Interproscan.sh version 5.68-101.0 and MetaboAnalyst version 6.0.

For manuscripts utilizing custom algorithms or software that are central to the research but not yet described in published literature, software must be made available to editors and reviewers. We strongly encourage code deposition in a community repository (e.g. GitHub). See the Nature Portfolio guidelines for submitting code & software for further information.

Data

Policy information about availability of data

All manuscripts must include a data availability statement. This statement should provide the following information, where applicable:

- Accession codes, unique identifiers, or web links for publicly available datasets
- A description of any restrictions on data availability
- For clinical datasets or third party data, please ensure that the statement adheres to our policy

The transcriptome datasets obtained from NCBI repository are from Bioproject: PRJNA544863 and PRJNA508937. Lipoxygenases sequences for phylogeny analysis were also obtained from NCBI and access codes are provided in the Supplemental Information. For all other datasets availability statement does not apply.

Research involving human participants, their data, or biological material

Policy information about studies with human participants or human data. See also policy information about sex, gender (identity/presentation), and sexual orientation and race, ethnicity and racism.

Reporting on sex and gender	N/A
Reporting on race, ethnicity, or other socially relevant groupings	N/A
Population characteristics	N/A
Recruitment	N/A
Ethics oversight	N/A

Note that full information on the approval of the study protocol must also be provided in the manuscript.

Field-specific reporting

Please select the one below that is the best fit for your research. If you are not sure, read the appropriate sections before making your selection.

☐ Life sciences ☐ Behavioural & social sciences ☒ Ecological, evolutionary & environmental sciences

For a reference copy of the document with all sections, see nature.com/documents/nr-reporting-summary-flat.pdf

Ecological, evolutionary & environmental sciences study design

All studies must disclose on these points even when the disclosure is negative.

Study description	Symbiotic and aposymbiotic individuals of the cnidarian sea anemone Aiptasia and cell cultures of the symbionts Breviolum minutum and Durusdinium trenchii were used as models for evaluating the effects of symbiosis and symbiont species identity in the cnidarian-dinoflagellate symbiosis. Symbiosis factor in the anemones was evaluated with two levels: aposymbiotic and symbiotic and in the symbionts: cultured and in hospite. Symbiont species identity factor for symbiotic anemones also had two levels: B. minutum and D. trenchii. More details are described in the Materials and Methods section.
Research sample	Symbiotic and aposymbiotic (i.e., symbiont free) individuals of Exaiptasia diaphana (Aiptasia, culture ID: NZ1) were used. For each phenotype there were N=3 replicate jars containing N=10 anemones in each that were pooled for the oxylipin analysis.<br><br>For the Symbiodiniaceae cell cultures (Breviolum minutum, culture ID CCMP830; Durusdinium trenchii culture ID D1A001), 150mL of cultures (N=3) of each species were collected. More details are provided on the Materials and Methods section.
Sampling strategy	No sample size calculation was performed.
Data collection	Data collection only applies to photosynthetic health measurements performed on sampling day as described in the Experimental setup of Materials and Method section. Measurements were performed by Marina T Botana using a Diving Pulse Amplitude Modulated Fluorometer (Diving-PAM, Walz, Effeltrich, Germany; settings: measuring light = 4, saturation intensity = 8, saturation width = 0.8 s, gain = 3, and damping =3), as described in the Materials and Methods section.
Timing and spatial scale	Aliquots for Symbiodiniaceae cell density measurements were sampled at days 0, 6, 12, and 16 of culture growth. On day 16, when cultures reached exponential growth phase, additional aliquots were collected for oxylipin analysis. Detailed sampling and analytical procedures are described in the Materials and Methods section.
Data exclusions	No data exclusion was performed.
Reproducibility	N/A
Randomization	N/A
Blinding	N/A

Did the study involve field work? ☐ Yes ☒ No

Reporting for specific materials, systems and methods

We require information from authors about some types of materials, experimental systems and methods used in many studies. Here, indicate whether each material, system or method listed is relevant to your study. If you are not sure if a list item applies to your research, read the appropriate section before selecting a response.

Materials & experimental systems

n/a	Involved in the study
☒	☐ Antibodies
☒	☐ Eukaryotic cell lines
☒	☐ Palaeontology and archaeology
☐	☒ Animals and other organisms
☒	☐ Clinical data
☒	☐ Dual use research of concern
☒	☐ Plants

Methods

n/a	Involved in the study
☒	☐ ChIP-seq
☒	☐ Flow cytometry
☒	☐ MRI-based neuroimaging

Animals and other research organisms

Policy information about studies involving animals; ARRIVE guidelines recommended for reporting animal research, and Sex and Gender in Research

Laboratory animals	Exaiptasia diaphana (Aiptasia, culture ID: NZ1), Symbiodiniaceae cultures (Breviolum minutum, culture ID CCMP830; Durusdinium trenchii culture ID D1A001).
Wild animals	N/A
Reporting on sex	N/A
Field-collected samples	N/A
Ethics oversight	N/A

Note that full information on the approval of the study protocol must also be provided in the manuscript.

Plants

Seed stocks	N/A
Novel plant genotypes	N/A
Authentication	N/A
